# From rare events to systematic data collection: the RESCUED registry for sudden cardiac death in the young in Germany

**DOI:** 10.1007/s00392-024-02460-z

**Published:** 2024-05-15

**Authors:** Renaldas Barkauskas, Tina Jenewein, Stefanie Scheiper-Welling, Verena Wilmes, Constanze Niess, Silvana Petzel-Witt, Alexandra Reitz, Elise Gradhand, Anastasia Falagkari, Maria Papathanasiou, Reza Wakili, David M. Leistner, Jessica Vasseur, Jens Göbel, Holger Storf, Stefan W. Toennes, Matthias Kettner, Marcel A. Verhoff, Britt-Maria Beckmann, Silke Kauferstein, Eva Corvest

**Affiliations:** 1 Centre for Sudden Cardiac Death and Familial Arrhythmias (CSCD), Institute of Legal Medicine, University Hospital Frankfurt, Goethe-University, Frankfurt/Main, Germany; 2 Department of Forensic Medicine, Institute of Legal Medicine, University Hospital Frankfurt, Goethe-University, Frankfurt/Main, Germany; 3 Department of Forensic Toxicology, Institute of Legal Medicine, University Hospital Frankfurt, Goethe-University, Frankfurt/Main, Germany; 4 Dr. Senckenberg Institute of Pathology and Human Genetics, University Hospital Frankfurt, Goethe-University, Frankfurt/Main, Germany; 5 DZHK (German Centre for Cardiovascular Research), Partner Site Rhein-Main, Frankfurt, Germany; 6 Institute of Medical Informatics, University Hospital Frankfurt, Goethe-University, Frankfurt/Main, Germany; 7 Department of Internal Medicine and Cardiology, University Heart Center, University Hospital Frankfurt, Goethe-University, Frankfurt/Main, Germany

**Keywords:** Sudden cardiac death (SCD), Registry, Heart, Cardiogenetics, Arrhythmia

## Abstract

**Background:**

Approximately one-third of sudden cardiac deaths in the young (SCDY) occur due to a structural cardiac disease. Forty to fifty percent of SCDY cases remain unexplained after autopsy (including microscopic and forensic-toxicological analyses), suggesting arrhythmia syndromes as a possible cause of death. Due to the possible inheritability of these diseases, blood relatives of the deceased may equally be carriers of the causative genetic variations and therefore may have an increased cardiac risk profile. A better understanding of the forensic, clinical, and genetic data might help identify a subset of the general population that is at increased risk of sudden cardiac death.

**Study design:**

The German registry RESCUED (**RE**gistry for **S**udden **C**ardiac and **U**n**E**xpected **D**eath) comprises information about SCDY fatalities and clinical and genetic data of both the deceased and their biological relatives. The datasets collected in the RESCUED registry will allow for the identification of leading causes of SCDY in Germany and offer unique possibilities of scientific analyses with the aim of detecting unrecognized trends, risk factors, and clinical warning signs of SCDY. In a pilot phase of 24 months, approximately 180 SCDY cases (< 50 years of age) and 500 family members and clinical patients will be included.

**Conclusion:**

RESCUED is the first registry in Germany collecting comprehensive data of SCDY cases and clinical data of the biological relatives reviewed by cardiac experts. RESCUED aims to improve individual risk assessment and public health approaches by directing resources towards early diagnosis and evidence-based, personalized therapy and prevention in affected families.

Trial registration number (TRN): DRKS00033543.

**Graphical abstract:**

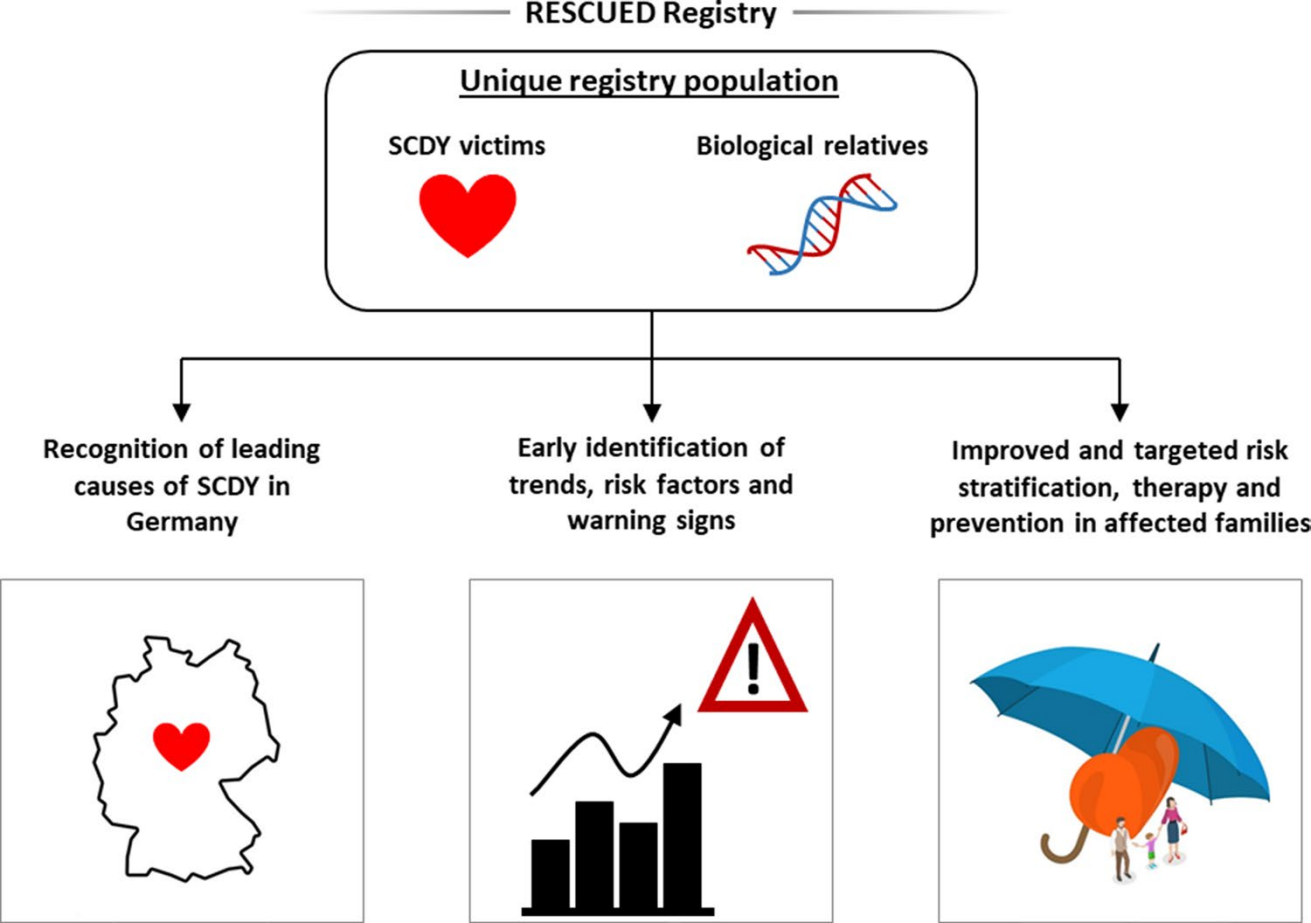

## Background

Approximately 1000–2000 people under 40 years of age die suddenly and unexpectedly per annum in Germany from cardiac causes [1]. These cases are referred to as sudden cardiac death in the young (SCDY). According to previous studies, a significant proportion of all SCDY occur due to a previously undiagnosed underlying genetic cardiac disorder, meaning that blood relatives of the deceased may equally be carriers of the causative genetic variation and therefore might be at an increased risk to also suffer a sudden cardiac death (SCD) [2]. The causes of SCD vary depending on the individual’s age. In the population under 40 years of age, cardiomyopathies (i.e., hypertrophic cardiomyopathy, dilated cardiomyopathy, and arrhythmogenic cardiomyopathy) are mainly responsible for SCDY. However, in an even younger cohort (i.e., pediatric patients and young adults), primary arrhythmogenic disorders (e.g., Long-QT-Syndrom (LQTS), Brugada Syndrome (BrS), and catecholaminergic polymorphic ventricular tachycardia (CPVT)) are mainly causative [3]. Thus, in an SCDY case, the objective is to unveil a potential genetic predisposition of the victim, and at the same time enable early identification of an inherited arrhythmogenic disease by clinical and — if needed — genetic testing of the victim’s biological relatives. Therefore, forensic pathologists and cardiologists play an important role in the prevention of sudden cardiac death in affected families.

Despite SCD increasingly gaining global awareness, systematic data collection in the form of registries is sparse. To the authors’ knowledge, to date, less than 20 registries recording SCD cases have been established worldwide, each with individually defined study designs and aims [4]. The World Health Organization defines SCD as “death within one hour of symptoms or within 24 h of last being seen well” [5]. While the broadness of this definition may be deliberate and necessary to encompass the myriad of clinical scenarios in which SCD may occur, it also allows for varying inclusion criteria in the different registries, making transferability and extrapolation of data and insights challenging. The latter is further complicated by variations in SCD incidence rates between different countries [6], suggesting the utility of national registries for obtainment of reliable data applicable to local demographics.

In Germany, the registry “SCD Germany” monitors and records sports-related SCD occurrences [7]. Sudden cardiac arrests (SCA) are documented in the German Resuscitation Registry (GRR), which includes both successful and futile resuscitation outcomes and has a clearly defined clinical focus on the chain of survival, treatment, and outcome rather than underlying causes of SCD [8]. Recently, the German Cardiac Arrest Registry (G-CAR) has been established, where, equally, the clinical aspects of SCA cases are in the spotlight, with a particular focus on cardiology and long-term outcomes [9].

**Fig. 1:**
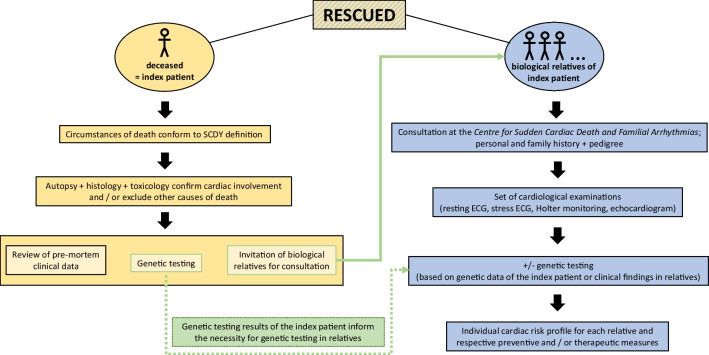
The RESCUED registry contains data from both the SCDY victim and their biological relatives. The workflow is oriented on international guidelines for SCD cases. First, the fatality is followed up to confirm the suspicion of SCDY and exclude other causes of death. Further, genetic testing of the SCDY victim is performed and their biological relatives are invited for a consultation at the Centre for Sudden Cardiac Death and Familial Arrhythmias, where a thorough personal and family history are recorded and a set of cardiological examinations is initiated. Whether biological relatives undergo genetic testing depends on their medical history, the clinical cardiological exams, and the results of the genetic tests in the index patient. Abbreviations: RESCUED, Registry for Sudden Cardiac and Unexpected Death; SCDY, Sudden Cardiac Death in the Young; ECG, electrocardiogram

A multidisciplinary registry collecting post-mortem data (i.e., autopsy with histology, toxicology, cardiopathology, and genetic data), medical history, and relevant clinical data before death, as well as clinical and genetic data of the victim’s family, is still missing. RESCUED (**RE**gistry for **S**udden **C**ardiac and **U**n**E**xpected **D**eath) is Germany’s first registry with a holistic, pedigree-based approach to SCDY cases. Apart from thoroughly documenting the cardiac deaths of young individuals under the age of 50, including their medical history and the results of their post-mortem genetic testing for variations in disease-associated cardiac genes, their relatives’ medical history, cardiac symptoms, and genetic testing results are equally recorded (Fig. 1). This multidisciplinary approach is one of the strong points of RESCUED.

Key aims of the RESCUED registry are to achieve a better understanding of SCDY through the identification of leading causes of SCDY in Germany and the early recognition of trends, risk factors, and warning signs of SCDY in order to optimize structures for appropriate nationwide clinical management and prevention strategies in families affected by SCDY.

## Study design

**Fig. 2:**
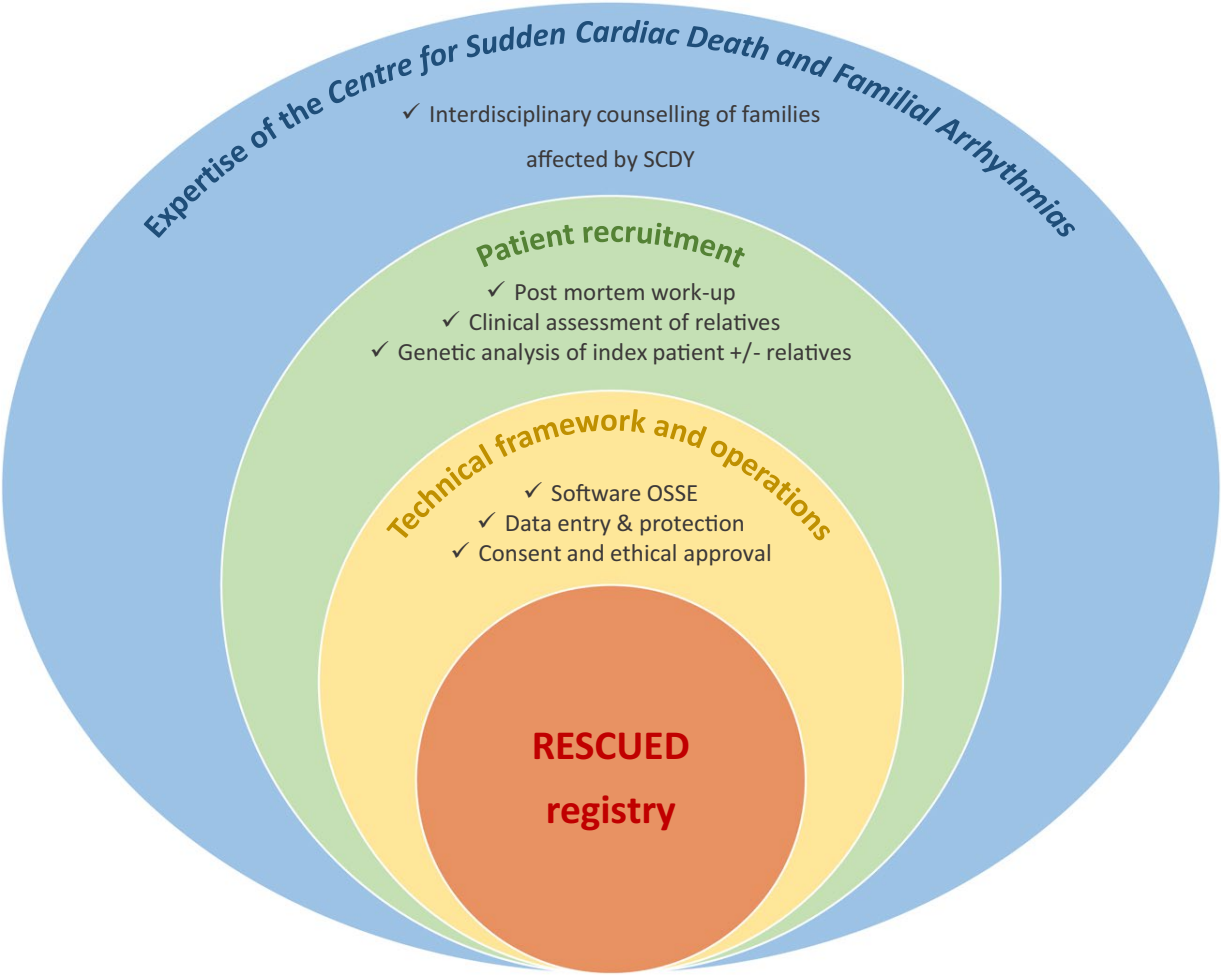
Expertise and structured efforts on multiple levels were necessary for the conception and development of the RESCUED registry. Through years of interdisciplinary counselling of families affected by SCDY involving forensic and clinical pathologists, cardiologists, and geneticists, we have established workflows based on international guidelines for post-mortem examination of SCDY victims and clinical assessment of their relatives. This practical experience with these complex cases laid the ground for the content of the registry, i.e., relevant information to be documented in order to achieve the desired outcomes. The technical and operational aspects involving software and data entry, as well as considerations regarding data protection and ethics, completed the framework for the conception of the RESCUED registry. Abbreviations: RESCUED (Registry for Sudden Cardiac and Unexpected Death); SCDY (Sudden Cardiac Death in the Young)

The design and composition of the RESCUED registry are the result of a complex set of forensic and clinical case work and technical and operational efforts on the backdrop of the interdisciplinary expertise of the Centre for Sudden Cardiac Death and Familial Arrhythmias (CSCD) at the University Hospital Frankfurt (Fig. 2).

Due to the vital need in Germany for such a registry, which bridges the link between the fields of forensics and cardiology, the German Cardiac Society (DGK) and the German Heart Foundation, as well as the German Society of Legal Medicine (DGRM), have agreed to endorse the project. RESCUED is listed in the German Clinical Trials Registry (DRKS) (Trial No. DRKS00033543), on the European Platform on Rare Disease Registration (ERDRI) and in the registry database of the German Institute for quality and patient safety (BQS).

### Target parameters

Based on our experience with complex cases of SCD and an in-depth literature research regarding SCD registries, we have developed designated questionnaires for systematic data collection. The RESCUED Registry contains two types of questionnaires: one for the deceased individual (index patient) and one for their biological relatives. Both questionnaires are subdivided into several forms as outlined in Tables 1 and 2. The questionnaire of the index patient can be linked to (multiple) questionnaires of their family members; this constitutes a “family-cluster,” which allows for all members of one family to be identified as biological relatives.

**Table 1:** The questionnaire for the deceased index patient in the RESCUED registry consists of eight forms (left column). Each form contains a carefully selected set of prompts and questions addressing the area of interest (right column). Shown is a selection of contents for each form

Questionnaire deceased index patient	
Form	Contents (selection)
1. Personal Data	Unique identifier (RESCUED-Number), biological sex, ethnicity, age at death
2. Circumstances of death	Observed event? Activity at time of death? Stimuli before death? Resuscitation? Decomposition?
3. Autopsy	Type of autopsy (clinical / forensic), body height, body weight, heart weight (cardiomegaly?), heart measurements, macroscopic cardiac and relevant non-cardiac findings
4. Further post-mortem analyses	Toxicology, histology, microbiology / virology (myocarditis?)
5. Cardiogenetic analyses	Biological material used for DNA extraction (type, quality?), type of analysis, variants in (cardiac) genes? Consanguinity in the family?
6. Medical history	Cardiac conditions (coronary, valvular, arrhythmogenic, ion channel disorders, cardiomyopathies) Cardiac symptoms over life time (dizziness, syncopes, seizures, dyspnea, palpitations, fever) Cardiac symptoms 24 h before death (dizziness, syncopes, seizures, dyspnea, palpitations, fever) ECG abnormalities (brady-/tachycardia, arrythmia, extrasystoles, QT-interval alterations) Non-cardiac conditions (vascular, pneumological, neurological, endocrinological, psychiatric/psychological) Medication (prescription / non-prescription)
7. Cause of death	Determined (yes/no), confirmed cardiac (yes/no), specification
8. Family history / pedigree	Biological relatives (yes/no)? Cardiological / cardiogenetic follow-up of relatives? Other SCDY cases in the family?

**Table 2:** The questionnaire for the biological relatives of the deceased index patient in the RESCUED registry consists of four forms (left column). Each form contains a carefully selected set of prompts and questions addressing the area of interest (right column). Shown is a selection of contents for each form. For each relative, a separate questionnaire is filled out and filed in the registry

Questionnaire biological relative of index patient	
Form	Contents (selection)
1. Personal Data	Unique identifier (RESCUED-Number), biological sex, ethnicity, age at the time of registry entry
2. Family history / pedigree	Biological relation to index patient, placement in the pedigree, family history at large with a focus on cardiac pathologies
3. Medical history	Cardiac conditions (coronary, valvular, arrhythmogenic, ion channel disorders, cardiomyopathies) Cardiac symptoms (dizziness, syncopes, seizures, palpitations, dyspnea, fever) ECG abnormalities (brady-/tachycardia, arrythmia, extrasystoles, QT-interval alterations) Non-cardiac conditions (vascular, pneumological, neurological, endocrinological, psychiatric/psychological) Medication (prescription / non-prescription)
4. Cardiogenetic analyses	Cardiogenetic testing performed (yes / no?), type of analysis, variants in (cardiac) genes? Consanguinity in the family?

All forms are available in both German and English language in order to lay the ground for potential international collaborations in future.

### Index patient

For the deceased index patient, the questionnaire is divided into eight forms (Table 1).

The information collected regarding the circumstances of death is designed to give a comprehensive understanding of the individual situation surrounding the event with a deliberate focus on important factors relevant in the context of SCD, such as activity at the time of death (strenuous physical activity, activities of daily life, sleep, etc.) or stimuli (visual, auditive, emotional). Certain stimuli can act as potential triggers of cardiac complications in patients with particular genetic predispositions (e.g., sudden loud noises can lead to cardiac arrhythmias in patients with long QT syndrome type 2 (LQTS2) [10]). The design of the RESCUED registry allows for the recognition and documentation of even such seemingly minute factors, creating a multipurpose dataset as a pre-requisite for a thorough analysis and evaluation of specific circumstances in SCD cases.

The forms documenting findings at autopsy and in histological, toxicological, and, where appropriate, microbiological/virological examinations consist of a balanced mix of targeted questions regarding cardiac findings, while also giving room for the documentation of any other noteworthy discovery.

In the form for cardiogenetic analyses, genetic variations found in genes associated with cardiac disorders are recorded regardless of the classification of the particular variant according to current ACMG (American College of Medical Genetics) consensus guidelines for interpretation regarding the pathogenicity of genetic variants in clinical testing. The rationale behind this inclusive recording of genetic variations in disease-associated cardiac genes in the RESCUED registry is the constant evolvement of knowledge in the field of (cardio) genetics, with the possibility of re-classification of certain genetic variants over time and the need for reassessment of such cases.

The form recording the medical history of the deceased person has a clear focus on cardiac conditions and events during the lifetime, but also specifically in the 24-h window before death. Equally, symptoms related to cardiac conditions are noted, including less known or obvious ones, such as fever, which could be indicative of myocarditis but also a trigger for arrhythmias, e.g., in patients with channelopathies such as LQTS or BrS [11], or seizures, which can occur as a consequence of cardiac arrhythmias. Medication is clustered into therapeutic groups (e.g., antiarrhythmic drugs, analgetics, antipsychotics), with a blank field provided for further specification.

### Biological relatives

The questionnaire in the RESCUED registry designated to record information about a biological relative of the deceased index patient includes four forms for data collection (Table 2).

When documenting the family history, it is important to gain an overall understanding of health and disease in the particular family, with a focus on cardiovascular pathologies. Placement of the individual within the pedigree is established and recorded, as well as their biological relation to the index patient who suffered an SCDY.

The individual’s medical history is documented in the respective form based on a thorough anamnesis and, where necessary, with the informed consent of the patient, the inspection of previous medical records, e.g., obtained from their general practitioner. Many of the prompts and questions in this form are identical to those in the index patient’s questionnaire, e.g., regarding symptoms, which may be indicative of cardiac conditions and may represent warning signs of previously undiagnosed cardiac abnormalities. Within the framework of the consultation to establish the individual’s cardiac risk profile in line with current clinical guidelines [12, 13], the patient undergoes a set of clinical cardiological examinations, including a resting ECG, a stress ECG, Holter monitoring, and an echocardiogram. Results of these tests are recorded in the RESCUED registry in the medical history form, and they are an important component in the overall assessment of the patient.

The question whether genetic testing is performed in a biological relative of the deceased index patient is complex, and the form documenting potential cardiogenetic test results needs to cater for this, taking into account all possible scenarios. The patient may not undergo any cardiogenetic testing, if:

- There is no evidence for pathological clinical findings in this patient and/or
- No clinically relevant variants in disease-associated cardiac genes have been discovered in the deceased index patient or
- They do not consent to genetic tests being performed.

If relatives do consent and the index patient carries a (likely) pathogenic variant in a cardiac gene, a cascade screening in the family will be performed. Further scenarios include the discovery of a variant of unknown significance (VUS) in a disease-associated cardiac gene in the index patient, leading to a complex assessment leaning on the medical history, cardiac symptoms, and possible warning signs of the living relatives to determine further proceedings. In this context, it is important to proceed with caution as to avoid unnecessary fearmongering within the family of an SCDY victim. The responsibility here lies with the medical team who must decide how to proceed in light of the obtained genetic testing results (VUS) of the index patient. All consultations in our outpatient clinic are carried out in accordance with the German Genetic Diagnostics Act (Gendiagnostikgesetz; GenDG) by our specialized cardiologist who also holds a qualification in cardiogenetic counselling.

## Patient recruitment

The RESCUED registry will retrospectively include all eligible cases of SCDY (aged < 50 years) examined in the CSCD at the Institute of Legal Medicine, University Hospital Frankfurt, Germany, and will continue to prospectively include such cases. SCDY cases are referred to the center from all over Germany mainly through institutes of legal medicine and cardiological centers.

Inclusion criteria (deceased):

- SCD at an age < 50 years
- Informed consent from next of kin

Exclusion criteria (deceased):

- Other natural or non-natural cause of death (e.g., lethal intoxication)
- Heavily decomposed cadavers
- Mechanical destruction of the myocardium
- No informed consent from next of kin

Inclusion criteria (relatives):

- Biological relative of a person who died of SCD at an age < 50 years
- Informed consent of the proband (or of the legal guardian if indicated)

Exclusion criteria (relatives):

- No informed consent of the proband (or of the legal guardian if indicated)

Uncertain cases are reviewed and discussed for eligibility to be entered into the RESCUED registry by an interdisciplinary panel comprising forensic and clinical pathologists, cardiologists, and geneticists.

**Fig. 3:**
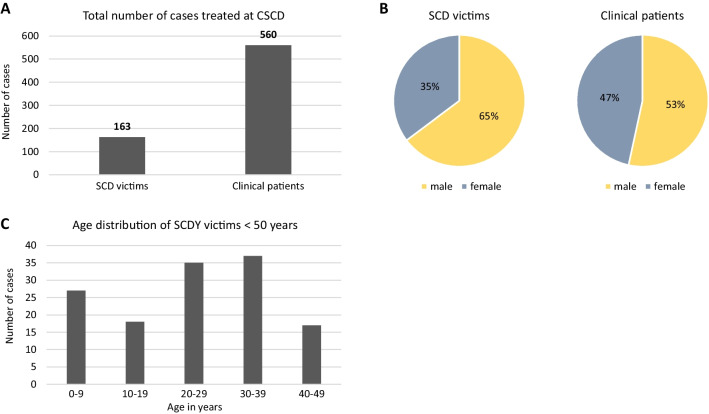
Our team at the CSCD, University Hospital Frankfurt, has treated the cases of 163 SCD victims and 560 clinical patients to date (**A**). While 2/3 of the SCD victims are male, the sex distribution is considerably more symmetrical in the clinical patient cohort (**B**). The age distribution among the SCDY victims under 50 years is rather balanced, with a slight peak in the third and fourth decade (**C**)

To date, we have collected 163 SCD cases and 560 clinical cases (predominantly biological relatives of SCD victims) from 13 federal states in Germany (Fig. 3A). The sex distribution of the SCDY cohort shows predominantly male victims, while the cohort of clinical patients is close to a 50:50 gender ratio (Fig. 3B). The age distribution of the SCDY victims under 50 years of age is fairly balanced, with a slight peak in the third and fourth decade (Fig. 3C).

### Post-mortem workflow

The post-mortem workflow in deceased individuals includes the review of the circumstances of death to ensure consistency with the WHO definition of an SCD (“death within one hour of symptoms or within 24 h of last being seen well”). Further, an autopsy, toxicological screening, and histological examination are performed to exclude other causes of death and/or confirm cardiac involvement. In the next step, DNA is extracted from material obtained at autopsy (e.g., EDTA blood/fresh frozen tissue/formalin-fixed paraffin-embedded tissue (FFPE)) for next-generation sequencing (NGS) and analysis of a panel of approximately 180 disease-associated cardiac genes. In parallel, pre-mortem clinical data are reviewed (i.e., underlying health conditions, ECG records, medication, etc.) to collect relevant information about the medical history of the deceased, with a particular focus on cardiovascular warning signs and events (Fig. 1).

### Clinical assessment and counselling of relatives

Biological relatives are invited for a consultation in our outpatient clinic at the CSCD to see our cardiologist specialized in rhythmology and cardiogenetics. Here, each individual’s personal medical history as well as the overall family medical history is compiled and a pedigree is created. Relatives are advised to undergo a set of cardiological examinations (resting ECG, stress ECG, Holter monitoring, and echocardiogram) which are then carefully examined by our specialized cardiologist. Depending on the constellation of clinical symptoms and data as well as genetic testing results of the deceased index patient, patients are offered genetic testing for the identified sequence variations in cardiac gene(s). The individual cardiac risk profile is established and, where needed, patients are advised regarding guideline-oriented preventive measures and therapeutic options (Fig. 1).

## Study organization

The RESCUED registry comprises a case review panel including cardiologists, geneticists, forensic pathologists, and cardiopathologists. Additionally, a steering committee with a chair-person and four additional members will be set up as the strategic decision-making body of RESCUED.

The RESCUED registry is based on OSSE (Open Source Registry System for Rare Diseases) [14], an open-source framework for setting up disease-specific patient registries without prior IT knowledge. The software is being developed, provided, and managed by the Institute of Medical Informatics (IMI) at the Goethe University Frankfurt. During the setup of the registry, data elements were defined in the central metadata repository and combined into electronic data entry forms according to the study design (Table 1). Staff of the CSCD enter the data obtained from the deceased and their biological relatives in the process described above (“Post-mortem workflow” and “Clinical assessment and counselling of relatives” and Fig. 1) into the RESCUED registry manually via the web-based interface of OSSE [14]. In addition to data validation checks during data entry based on validation rules from the central MDR, data is verified and cleared by at least one senior researcher using the form status workflow provided by OSSE.

The RESCUED registry is designed to comply with all relevant data protection requirements as specified in the registry’s data protection concept. Data is pseudonymized during data entry using the web-based open-source pseudonymization service provided by the Johannes Gutenberg-University Mainz, the so-called Mainzelliste [15]. An individual’s identifying data is stored separately from the medical data in the database of the Mainzelliste, which creates pseudo-identifiers for use in the OSSE database. In addition, access to the data stored in the RESCUED registry is controlled via the built-in modular role concept of OSSE.

Funding for the conception of the RESCUED registry was obtained from the German Heart Foundation. Independently of the RESCUED trial, all post-mortem and clinical examinations of the SCDY victims and clinical patients are covered either by state funds, health insurance funds, or research funds.

## Consent and ethics statement

Overall ethical approval for the RESCUED registry was obtained from the Ethics Committee of the Medical Faculty of the University of Frankfurt, Germany, on 13.11.2023 (Project Number 136/23).

Written consent for the post-mortem work-up including genetic testing in SCDY victims and the entry of anonymized data into the RESCUED registry is obtained from the next-of-kin. Adult and competent relatives consent for themselves. For minors and/or persons incapable of consent, written consent is obtained from the legal guardians. All participants have the right to retract their consent and request the deletion of their data from the RESCUED registry at any time point.

## Data and statistical analysis

For this study, descriptive statistics will mainly be used. Data will be expressed as means ± SD. Regression models will be used to identify patients’ characteristics that are associated with a defined phenotype/genotype. The significance level for the alpha error was set at *p* < 0.05. Based on the explorative character of the study, statistical assessment is possible at any time.

## Discussion

SCDY events fortunately remain rare, the flip side being that expertise regarding the cardiological, pathological, and genetic aspects of SCDY is scattered and interdisciplinary structures to ensure a guideline-oriented follow-up of affected families are rarely in place. For the characterization of SCD on a population level, capturing as many cases as possible is crucial. The usage of multiple sources and the integration of such multitudinal data is a key distinguishing feature of this registry.

RESCUED is Germany’s first registry with a holistic, pedigree-based approach to SCDY, born out of years of multidisciplinary experience. Our interdisciplinary team has established itself as a reliable point of contact to follow up on SCD cases reported to us by forensic or clinical pathologists or clinicians nationwide, and offer guidance and expert advice to relatives of the deceased. Over the course of time, we have recognized the necessity for a registry for the SCDY cases that we treated, because a structured collection of clinical, macro- and histopathological, and genetic data on SCDY victims and their families was lacking in Germany to this date.

### Post-SCDY diagnostics

Recent guidelines have focused on attendance to patients with sudden unexplained death and their families [12]. Several studies have shown that in reality, however, essential diagnostics as mentioned in the guidelines were often not performed. The European Heart Rhythm Association survey on the investigation on SUDY (sudden unexpected death in the young) revealed that, on average, less than half of SUDY cases are investigated with autopsy [16]. In addition, comprehensive post-mortem investigations (i.e., toxicology, histopathology) are not always performed [17].

Post-mortem genetic testing (molecular autopsy) has become an important tool when an inherited cardiac disease is suspected as the underlying cause of death. For instance, Bagnall et al. showed in their prospective study that, in unexplained SCD cases, autopsy investigations combined with histology, toxicology, and genetic testing revealed a likely etiology in 27% of cases. After follow-up of first-degree relatives, a definite clinical diagnosis was established in 13% [2]. These findings corroborate the importance of the post-SCDY diagnostics for the prevention of new SCD cases in the remaining family members. Thus, current guidelines and consensus documents recommend retaining samples and performing post-mortem genetic testing in SCDY cases which undergo autopsy including histological and toxicological examinations when an inherited cardiac disease is suspected [12, 18]. Despite many difficulties and challenges that come with performing structured investigations after an SCDY, our multidisciplinary expert team has developed and implemented a standardized post-SCDY diagnostic protocol (Fig. 1) to improve access to comprehensive genetic, clinical, and forensic services that align better with current recommendations for families affected by SCDY. The robust and standardized approach to data collection represents an important pillar of the RESCUED registry.

### Diagnosis prior to SCDY

While we have a laid-out strategy in patients with known heart diseases, we do not have any in patients without. This is an important issue, given that a substantial proportion of SCDY occur in patients without any diagnosed cardiac condition [19]. Thus, there is a need to move towards high yield, multi-parametric scores to improve the accuracy of prediction to identify the high-risk group in the general population, where the largest absolute numbers of SCD are encountered. This will emerge through data collection and the use of artificial intelligence, and represents one of the goals of this trial.

Contrary to general belief, a substantial proportion of SCD patients have previously been in contact with healthcare providers before their death. A recent study of sudden cardiac arrest in children revealed that 22% of the children had seen a pediatric cardiologist before the SCD event, while 9% had been diagnosed with a cardiac condition predisposing to SCD or an unrecognized cardiac condition (13%) [20]. The EndUCD trial [21] investigated out-of-hospital cardiac arrest (OHCA) in 1–50-year-old individuals on a multi-source data basis. Fifteen percent of the patients had a diagnosis prior to their OHCA and 8% either had been evaluated for risk factors or had symptoms investigated. These results suggest that not in all evaluated patients, an SCD risk can be recognized and even if an SCD risk was recognized, not all cardiac arrest can be prevented. In general, several studies demonstrated that SCD is preceded by symptoms in around half of the cases [22]. A nationwide study among sudden arrhythmic death in the young in Denmark identified cardiac symptoms in 35% of the patients, most commonly syncope. Among this group, only one out of five had had contact to the healthcare system. These findings emphasize the importance of a detailed evaluation of patients and the necessity to improve our knowledge of SCDY. For instance, we need to collect standardized, detailed information regarding characteristics and onset of symptoms and clinical investigations prior to death, which will inform the development of strategies for so-called near-term prevention, consisting of prompt action in response to warning signs. RESCUED will add crucial information of early warning signs and its association with the underlying heritable cardiac diseases, which would help to pre-empt SCDY by timely detection and intervention within a short period prior to death.

### Biological relatives of SCDY victim

SCDY is always a tragedy for those left behind and leads to significant anxiety in the family. Mainly two questions arise: why did my relative die suddenly and what risks apply to the other family members? Potential genetic cardiac diseases can be the underlying cause in 25–49% of SCD in the young (< 50 years of age). In addition to the fact that identifying the cause gives the family an explanation for the early death, it allows further screening of the family [13]. This creates the possibility of early diagnosis and thus enables personalized therapy and/or prevention. Recently, new guidelines for the management of patients with ventricular arrhythmias (VA) and the prevention of SCD have been published by the European Society of Cardiology (ESC) to improve the care of patients at risk [13]. The guidelines reinforce the importance of multidisciplinary teams and specialized centers for clinical and genetic testing (class I recommendation). Based on these recommendations, following an SCDY, family evaluation is encouraged in the majority of cases in which an inherited cardiac disease is suspected in the victim (class I). Clinical assessment of family members should include medical history, physical examination, ECGs, cardiac imaging, and exercise testing. Follow-up and periodic re-evaluation are important and are dictated by initial findings [12]. If a (likely) pathogenic genetic alteration is identified in the SCDY victim, cascade genetic analysis should be offered in relatives [13]. In the translation of these recommendations into clinical practice, first-line ECG and echocardiography are near ubiquitous, but the protocols appear to diverge, despite successive recommendations on exercise ECG and data supporting the greater sensitivity of high precordial lead ECGs for the detection of Brugada type 1 ECG pattern [16]. This may result in a misdiagnosis of the causes of SCDY and potentially put family members at increased risk. RESCUED will provide information on clinical data of the relatives of an SCDY victim, reviewed by a multidisciplinary team, based on current guidelines. This may help to avoid underreporting and misdiagnosis and will offer new insights for risk stratification and personalized therapy and prevention.

## Conclusion

Improvement and expansion of existing specialist structures are needed for the investigation of the causes of SCDY and identification of relatives at risk of SCD among remaining family members. With its structural and multidisciplinary approach, RESCUED will provide more insights for a better understanding of the causes and improve prevention strategies.

In summary, the RESCUED registry constitutes a novel platform on the landscape of attendance to families affected by an unexpected cardiac death in a person under the age of 50, currently operating on a national level in Germany, with future international collaborations evolving. Through a systematic approach to the documentation of SCDY cases, new observations and patterns might emerge and will offer new possibilities to contextualize e.g. possible specific preceding warning signs and symptoms of SCDY. Ultimately, insights gained from the RESCUED registry will lead to improved and targeted diagnostic possibilities, risk stratification, and, if need be, therapy in susceptible individuals, working towards the prevention of tragic deaths in affected families.

## Declarations

### Disclaimer

The RESCUED registry is an investigator-initiated project supported mainly by the German Heart Foundation (Deutsche Herzstiftung). The CSCD is further supported by the Dr. Hans Messer Foundation, the Dr. Rolf M. Schwiete Foundation, and the Friedrich Flick Foundation. The authors are solely responsible for the design, the composition, and the management of the registry as well as the drafting, editing, and the final contents of this publication. The abovementioned foundations have no influence on the registry design, data collection and the manuscript at hand.
